# Risk factors for checkpoint inhibitor pneumonitis in lung cancer patients treated with immune checkpoint inhibitors: a systematic review and meta-analysis

**DOI:** 10.3389/fimmu.2025.1607170

**Published:** 2025-05-21

**Authors:** Xiaoqing Zhou, Yingnan Xu, Yuanyuan Ying, Ruilin Chen, Zhen Wang, Xin Lv

**Affiliations:** ^1^ The First Affiliated Hospital of Zhejiang Chinese Medical University (Zhejiang Provincial Hospital of Chinese Medicine), Hangzhou, Zhejiang, China; ^2^ The First School of Clinical Medicine, Zhejiang Chinese Medical University, Hangzhou, Zhejiang, China

**Keywords:** meta-analysis, checkpoint inhibitor pneumonitis, immune checkpoint inhibitors, risk factor, immune-related adverse events

## Abstract

**Background:**

Immune checkpoint inhibitors (ICIs) significantly improve survival in lung cancer patients. However, checkpoint inhibitor pneumonitis (CIP) remains a critical safety concern. This meta-analysis systematically evaluates demographic, clinical, and laboratory risk factors associated with CIP development to guide risk-stratified management.

**Methods:**

We systematically searched eight databases from inception to February 20, 2025. Study quality was assessed using the NOS. Adjusted risk factors from multivariate analyses were pooled in RevMan 5.4. Sensitivity analyses addressed heterogeneity, and funnel plots evaluated publication bias.

**Results:**

28 NOS-certified high-quality studies were included. 20 risk factors comprised: advanced age, male sex, smoking status, preexisting interstitial lung abnormalities, pulmonary fibrosis, COPD, thoracic radiotherapy history, squamous cell carcinoma histology (versus adenocarcinoma), early-stage NSCLC (Stage III versus IV), multifocal metastases (≥2 sites), PD-1 inhibitors (versus PD-L1 agents), elevated PD-L1 expression (≥50%), pembrolizumab use (versus nivolumab), AEC, CRP, PLR, WBC, and hypoalbuminemia. Sensitivity analyses confirmed consistency between FEM and REM; funnel plots indicated no publication bias.

**Conclusion:**

This study identifies 20 independent CIP risk factors in ICI-treated lung cancer patients. Early screening and intervention for high-risk populations are critical to reducing CIP incidence and improving clinical outcomes. These findings provide actionable insights for optimizing ICI safety in real-world practice.

**Systematic review registration:**

https://www.crd.york.ac.uk/PROSPERO/myprospero, identifier CRD420250655469.

## 1 Introduction

Lung cancer remains a major global public health concern, characterized by persistently high incidence and mortality rates for malignant tumors (1). Despite significant therapeutic advancements, including surgical resection, platinum-based chemotherapy, radiotherapy, and molecular targeted therapies, the five-year survival rate for patients with advanced-stage disease remains below 17% (2). The introduction of immune checkpoint inhibitors (ICIs), such as anti-CTLA-4, anti-PD-1, and anti-PD-L1 agents, has led to a paradigm shift in oncology by restoring T cell-mediated antitumor immunity through the blockade of immune evasion pathways (3). These agents have significantly improved progression-free survival and overall survival in clinical trials (4). However, the systemic immune activation induced by ICIs can trigger a cascade of pathological events (1): an imbalance between Th17 and Treg cells that disrupts T-cell homeostasis (2), a cytokine storm driven by excessive production of IL-1β, TNF-α, and CXCL-10, and (3) the loss of self-tolerance, resulting in the production of autoantibodies (5). Epidemiological data underscore a concerning disparity in the incidence of checkpoint inhibitor pneumonitis (CIP). Clinical trials report a 3-5% CIP incidence, with associated mortality rates ranging from 10-17% (6). However, real-world studies indicate higher risks in specific patient subgroups. For example, a meta-analysis of 2,314 stage III NSCLC patients receiving durvalumab after chemoradiation reported a CIP incidence of 35% (7), highlighting the important role of treatment sequencing and prior thoracic radiotherapy.

The diagnosis of CIP is complicated by its nonspecific clinical and radiological features, which overlap with those of infectious pneumonia, lymphangitic carcinomatosis, tumor progression, and diffuse alveolar hemorrhage (8). As the most frequent and potentially fatal immune-related adverse event (irAE) in patients receiving PD-1/PD-L1 monotherapy (9), CIP significantly increases mortality risk. Current clinical protocols often require the permanent discontinuation of ICIs in most CIP cases. Although high-dose glucocorticoid therapy can induce clinical remission, rechallenging immunotherapy frequently leads to recurrent grade ≥2 pneumonitis, necessitating the permanent cessation of ICIs and impairing long-term antitumor treatment strategies (10). The pathogenesis of CIP remains poorly understood, emphasizing the urgent need to identify validated risk factors for early diagnosis and personalized management. Such efforts would help reduce unnecessary diagnostic interventions and mitigate tumor progression associated with treatment discontinuation (11). Various risk factors have been reported, including demographic characteristics, pre-existing pulmonary conditions, concurrent use of immunosuppressants (12, 13), history of radiotherapy (14), and hematologic biomarkers (15). However, these findings demonstrate significant inconsistencies across studies. To address these gaps, a systematic synthesis and quantitative evaluation of these factors is necessary, making a comprehensive meta-analysis essential for establishing evidence-based risk stratification frameworks.

## 2 Method

### 2.1 Registration review

The methodological framework and reporting standards were carefully designed and adhered to by the Preferred Reporting Items for Systematic Reviews and Meta-Analyses (16). The review was prospectively registered with PROSPERO (registration number: CRD420250655469) before the initiation of the systematic review process.

### 2.2 Literature sources and search strategy

A systematic, multistage search protocol was implemented across eight electronic databases, comprising four international biomedical repositories (PubMed, Web of Science, Embase, and Cochrane Library) and four Chinese scholarly databases (CNKI, Wanfang Data, VIP Journal Integration Platform, and CBM), covering the period from database inception to February 20, 2025. The search framework utilized controlled vocabulary (MeSH terms) and free-text keywords about the conceptual domains of “Lung Neoplasms” “Immune Checkpoint Inhibitors” “Pneumonia” and “Risk Factors”, with the PubMed search algorithm detailed in **Appendix 1**.

To ensure methodological rigor, three validation mechanisms were implemented (1): backward citation tracking of included studies’ reference lists, (2) forward citation searching via Web of Science, and (3) gray literature search through OpenGrey, ClinicalTrials, and the WHO International Clinical Trials Registry Platform. This tripartite approach met the PRISMA requirements for comprehensive evidence retrieval.

### 2.3 Selection criteria

Inclusion criteria: (1) study design: case-control or cohort studies; (2) study population: histologically confirmed lung cancer patients receiving PD-1/PD-L1 inhibitors either as monotherapy or in combination with CTLA-4 blockade; (3) diagnosis of CIP: radiological confirmation of CIP through characteristic high-resolution computed tomography (HRCT) findings (e.g., ground-glass opacities, consolidations) or histopathological verification; (4) grouping criteria: explicit comparison between CIP development cohorts and non-CIP controls; (5) effect estimates: reported multivariable-adjusted effect estimates, including OR or HR with 95% CIs.

Exclusion criteria: (1) Lung cancer patients not receiving ICIs; (2) Pneumonia attributed to other causes (e.g., infectious pneumonia, radiation pneumonitis, non-ICI drug-associated pneumonia); (3) Studies in which CIP was not analyzed independently of other irAEs; (4) Studies focusing solely on the safety and efficacy of ICIs without evaluating the relationship between risk factors and CIP; (5) Studies that only performed univariate analyses without correcting for confounders; (6) Incomplete reporting of effect sizes; (7) Total sample sizes <50 cases; (8) Conference abstracts, reviews, and case reports; (9) Low-quality, repetitive publications.

### 2.4 Data extraction and quality assessment

To ensure data accuracy and consistency, all information was independently extracted and cross-checked by two researchers (Xiaoqing Zhou and Yingnan Xu). The extracted data included study design, country, study type, sample size, risk factors, and statistical effects. In cases of disagreement, consensus was reached through discussion or, if needed, with input from a third researcher, Xin Lv, to maintain consistency and high-quality data extraction.

Additionally, Xiaoqing Zhou and Yingnan Xu independently assessed the quality of all included studies using the Newcastle-Ottawa Scale (NOS), which evaluates selection bias, comparability, and outcome/exposure. Studies scoring ≥7 out of 9 were considered high quality, while those with scores <7 were excluded from subsequent analyses. Discrepancies were resolved through discussion, with Xin Lv consulted when necessary to ensure the accuracy and fairness of the assessment.

### 2.5 Statistical analysis

Meta-analyses were performed on articles with two or more independent studies. Risk factors identified through multivariate analyses were stratified using RevMan 5.4. Pooled ORs with 95% CIs were calculated using inverse variance weighting. Heterogeneity was assessed with Cochran’s Q test and I² statistics. A fixed-effects model (FEM) was used for minimal heterogeneity (P > 0.05, I² < 50%), and a random-effects model (REM) was applied for significant heterogeneity (P ≤ 0.05, I² ≥ 50%). Sensitivity analyses were conducted by sequentially excluding studies, and subgroup analyses were based on study characteristics. Statistical significance was set at P < 0.05. Publication bias was assessed visually with funnel plots, with symmetry indicating minimal bias.

## 3 Results

### 3.1 Literature search results

**Figure 1:**
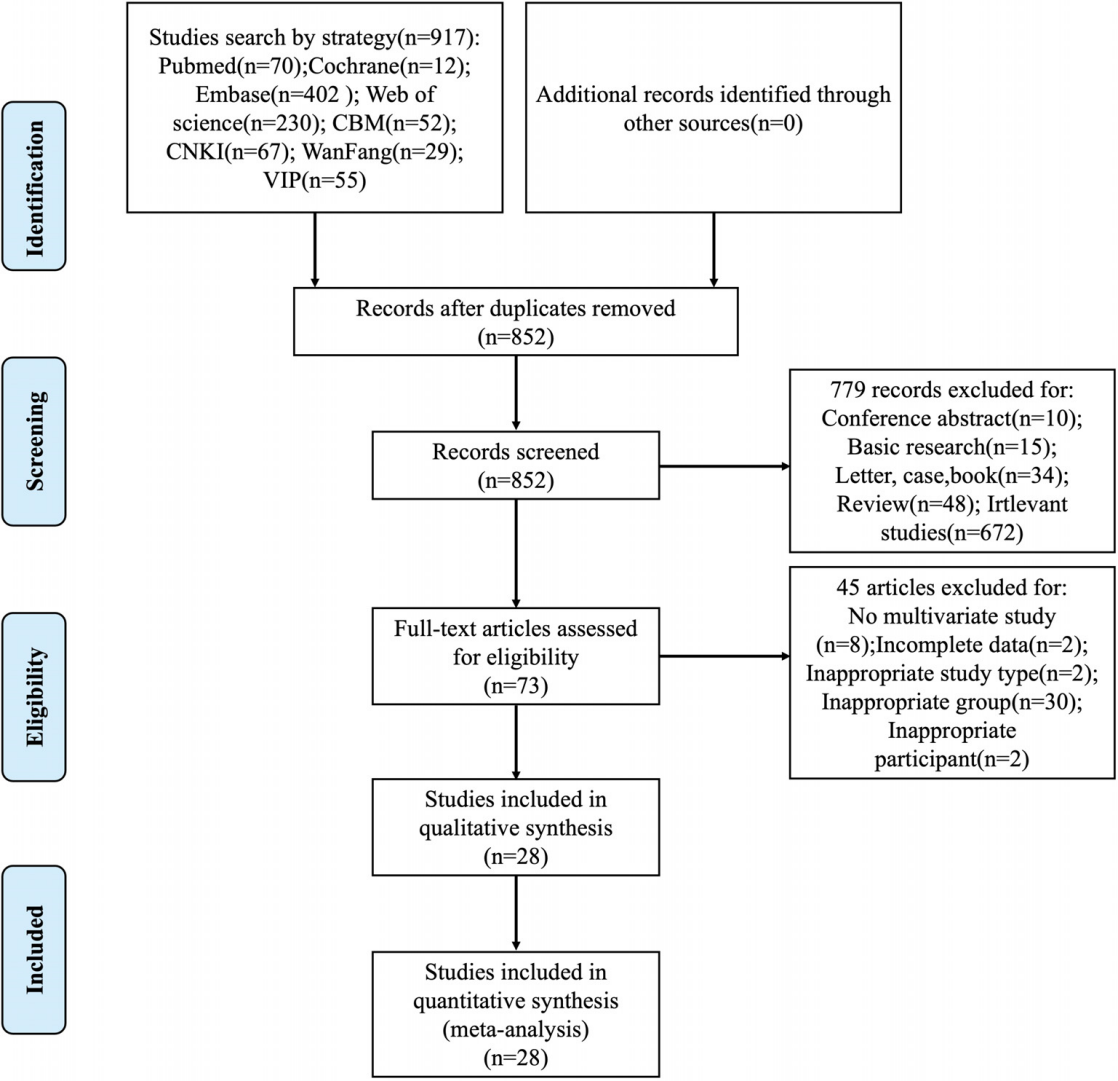
Flow diagram of the study selection.

The systematic literature retrieval identified 917 candidate publications, from which 852 unique records remained following duplicate removal. A preliminary review of the titles, abstracts, and keywords led to the exclusion of 779 articles, leaving 73 articles for full-text evaluation. Through rigorous application of predefined eligibility criteria, 45 studies were excluded based on specific exclusion criteria: absence of multivariate analysis implementation (n=8), incomplete parameter reporting (n=2), non-conforming study methodology (n=2), flawed cohort stratification (n=30), and ineligible patient eligibility criteria (n=2). The final analytical cohort comprised 28 high-quality studies that underwent formal meta-analytical integration, with the complete selection pathway delineated through the PRISMA-compliant flowchart in **Figure 1**.

### 3.2 Characteristics and quality assessment of included studies

The systematic review incorporated 28 eligible studies, including 20 case-control investigations (17–36) and 8 cohort analyses (37–44). These investigations collectively enrolled 1,075 CIP-diagnosed patients and 7,455 matched controls, with geographic distribution as follows: China contributed 50.0% of studies (14/28), followed by the United States (28.6%, 8/28), Japan (14.3%, 4/28), and South Korea (7.1%, 2/28). The publication timeframe encompassed research outputs from 2018 through 2025. Methodological evaluation via the Newcastle-Ottawa Scale revealed all studies achieved quality scores ≥7, confirming rigorous adherence to observational research standards. Complete demographic distributions and quality assessment metrics are cataloged in **Table 1**.

**Table 1:** Characteristics of the 28 studies included in the meta-analysis.

Study	Country	Types of study	Samples		NOS score	Risk factors(multivariate data)
			CIP	non-CIP		
2023 Cheng M (18)	China	Case-control study	32	81	9	6), 18), 44), 48), 50), 51)
2023 Zhang Y(1) (27)	China	Case-control study	42	484	9	1), 14), 18), 44), 48), 52)
2023 Zhang Y(2) (27)	China	Case-control study	18	208	9	1), 14), 18), 44), 48), 52)
2022 Jia X (20)	China	Case-control study	88	418	9	1), 2), 3), 8), 11), 12), 13), 21), 23), 24), 25), 26), 27), 28), 34), 42), 44), 45), 47), 48), 49), 50) 52), 53)
2022 Yamaguchi T (39)	Japan	Case-control study	17	108	8	1), 2), 8), 35)
2022 Wang H (7)	China	Case-control study	23	44	8	3), 56), 57)
2021 Isono T (23)	Japan	Case-control study	27	153	9	7), 10), 11), 31), 43), 46), 47), 52), 54)
2020 Chu X (24)	China	Case-control study	54	246	7	1), 2), 42)
2020 Moda M (25)	Japan	Case-control study	22	159	9	15), 19), 22), 36), 47
2020 Zhang C (19)	China	Case-control study	16	78	9	7), 18)
2019 Fukihara J (26)	Japan	Case-control study	27	143	8	32), 54)
2019 Shibaki R (37)	Japan	Cohort study	36	295	9	2), 3), 8), 19)
2018 Cho JY (38)	South Korea	Cohort study	22	145	9	1), 8), 29)
2018 Suresh K (17)	The United State	Case-control study	39	166	9	19)
2018 Yamaguchi T (39)	Japan	Cohort study	18	105	9	2), 7), 11)
2023 Wong A (40)	The United State	Cohort study	39	432	9	10), 15), 20), 30
2022 Sawa K (41)	Japan	Cohort study	33	295	9	1), 2), 3), 16), 19)
2021 Jung J (42)	South Korea	Cohort study	23	219	9	3), 20)
2021 Lin X (28)	China	Case-control study	87	87	8	14), 18), 19), 34)
2021 Atchley W T (29)	The United State	Case-control study	30	285	9	7), 9), 32), 33)
2023 Arai T (43)	Japan	Cohort study	23	150	9	1), 2), 3), 15), 18), 19), 22), 29), 30), 37)
2023 Altan M (30)	The United State	Case-control study	40	379	8	5), 8)
2024 Yang J (31)	China	Case-control study	35	194	9	2), 3), 6), 42), 45), 52), 58)
2024 Sumi T (32)	Japan	Case-control study	13	63	9	4), 7), 19), 59), 60)
2022 Chao Y (33)	China	Case-control study	20	144	9	9), 31), 55)
2025 Cui L (14)	China	Case-control study	102	652	9	8), 15), 30), 38)
2024 Li Y (52)	China	Cohort study	16	128	8	3), 9), 10), 15). 30)
2024 Hong B (35)	China	Case-control study	59	573	9	21), 23), 40), 41)
2024 Li X (52)	China	Case-control study	110	1021	8	2), 6), 17), 39)

Demographic features, 1) Age, 2) Sex (male versus female), 3) Smoking status. Clinical features, 4) Overall tumor burden ≥85 mm, 5) Shortness of breath, 6) Lung diseases, 7) Pulmonary fibrosis (PF), 8) Interstitial Lung Disease (ILD), 9) Chronic Obstructive Pulmonary Disease (COPD), 10) Interstitial Lung Abnormalities (ILA), 11) Emphysema, 12) Hypertension, 13) Diabetes, 14) Prior radiotherapy, 15) Prior thoracic radiotherapy, 16) Prior operation, 17) Prior antiangiogenic therapy, 18) Eastern Cooperative Oncology Group Performance Status (ECOG PS) (≥2 versus <2), 19) Histology (Squamous cell carcinoma versus Adenocarcinoma), 20) Stage III NSCLC vs IV, 21) Number of metastatic sites≥ 2, 22) Tumor invasion in the central airway, 23) Pulmonary metastasis, 24) Lymphatic metastasis, 25) Bone metastasis, 26) Hepatic metastases, 27) Brain metastases, 28) Adrenal metastasis, 29) Extrathoracic metastasis, 30) PD-1/PD-L1 immunotherapy, 31) PD-L1 expression status ≥50%, 32) Drug, Pembrolizumab vs Nivolumab, 33) Drug, Ipilimumab vs Nivolumab, 34) Combined treatment (Combined IO/IO monotherapy), 35) Chemotherapy (PEM vs. PTX/nab‐PTX), 36) Line of chemotherapy, 37) Treatment line, first, 38) Concurrent thoracic radiotherapy, 39) Concurrent chemotherapy, 40) Concurrent antibiotic, 41) Concurrent PPI. Laboratory features, 42) Absolute Eosinophil Count (AEC), 43) Eosinophils, 44) Absolute Lymphocyte Count (ALC), 45) Absolute Neutrophil Count (ANC), 46) Monocytes, 47) C-Reactive Protein (CRP), 48) Neutrophil-to-Lymphocyte Ratio (NLR), 49) Platelets (PLT), 50) Platelet-to-Lymphocyte Ratio (PLR), 51) Lactate Dehydrogenase (LDH), 52) White Blood Cell Count (WBC), 53) Systemic Immune-Inflammation Index (SII), 54) Low Albumin, 55) IL-8, 56) IL-10, 57) IL-12, 58) CD4+ T lymphocyte, 59) Percentage of Diffusing Capacity of the Lung for Carbon Monoxide (%DLCO), 60) Surfactant Protein D (SP‐D).

### 3.3 Meta-analysis results

**Figure 2:**
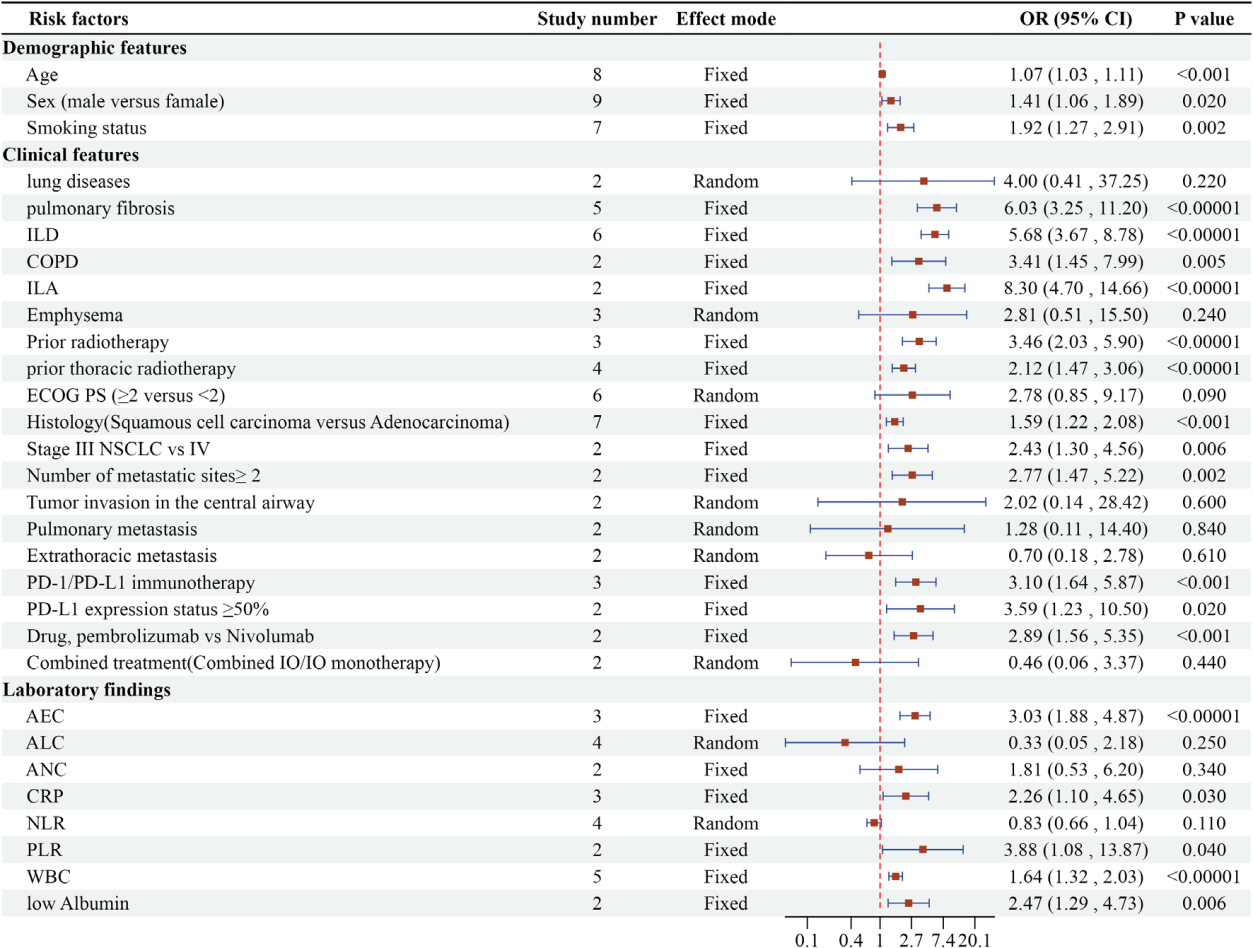
Meta-analysis results of multivariate analysis of CIP in ICI-treated lung cancer patients.

Quantitative synthesis identified 60 potential risk indicators across three domains: demographic parameters (n=3), clinical variables (n=38), and laboratory biomarkers (n=19). The subsequent analysis focused on 30 recurrent factors reported in ≥2 independent studies. Detailed information is presented in **Figure 2**.

#### 3.3.1 Demographic characteristics

**Figure 3:**
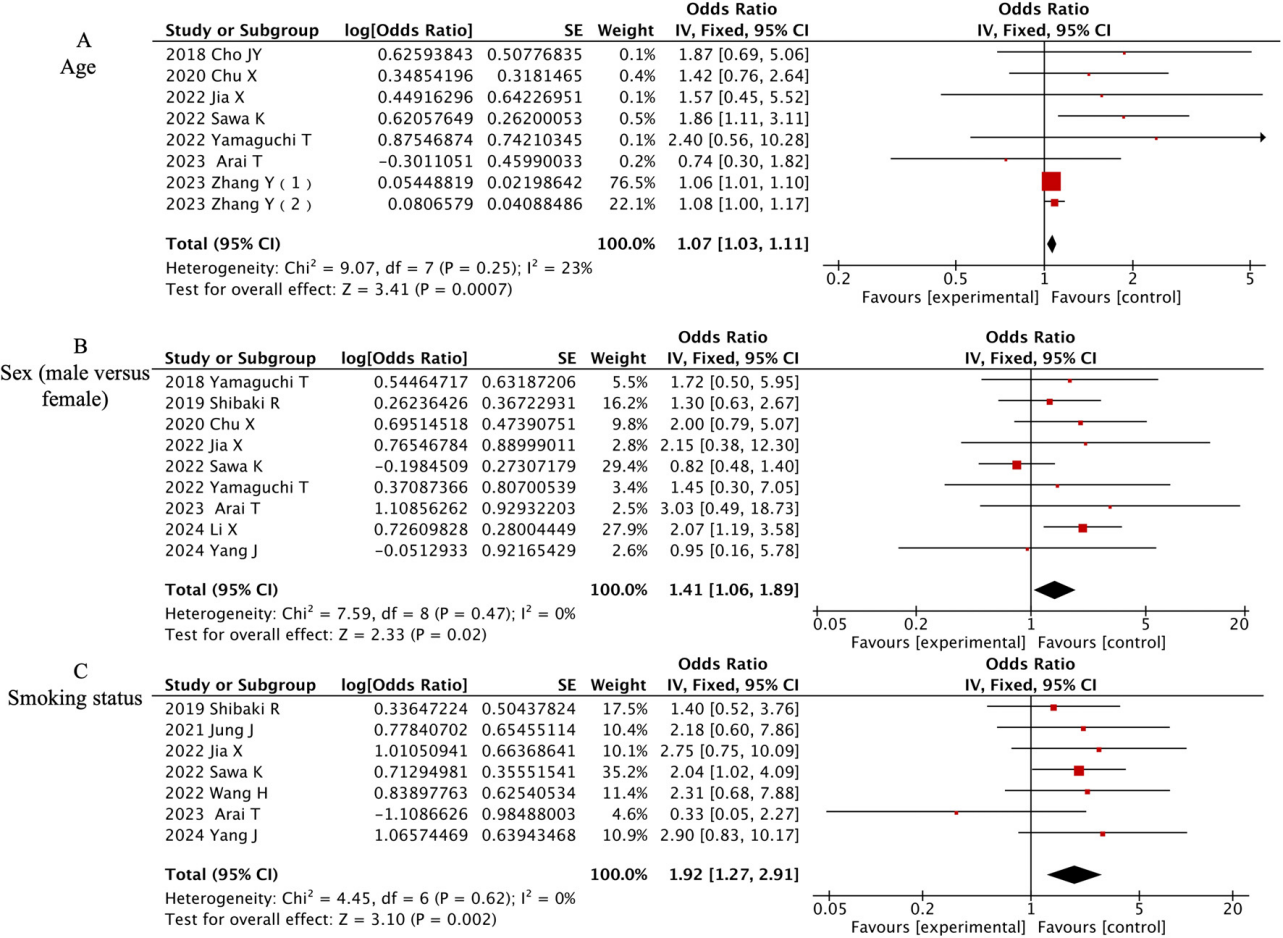
Forest plot of meta-analysis on significant demographic-associated risk factors for CIP in ICI-treated lung cancer patients. **(A)** Age, **(B)** Sex (male versus female), **(C)** Smoking status.

Three demographic variables demonstrated significant associations with CIP development in multivariate analyses. Detailed forest plots are presented in **Figure 3**. Advanced age emerged as an independent predictor across 8 studies (OR=1.07, 95% CI 1.03-1.11, P=0.0007). Male patients showed 41% higher CIP risk compared to females in 9 studies (OR=1.41, 95% CI 1.06-1.89, P=0.0007). Current or former smokers exhibited nearly doubled CIP risk relative to non-smokers in 7 investigations (OR=1.92, 95% CI 1.27-2.91, P=0.002).

#### 3.3.2 Clinical characteristics

**Figure 4:**
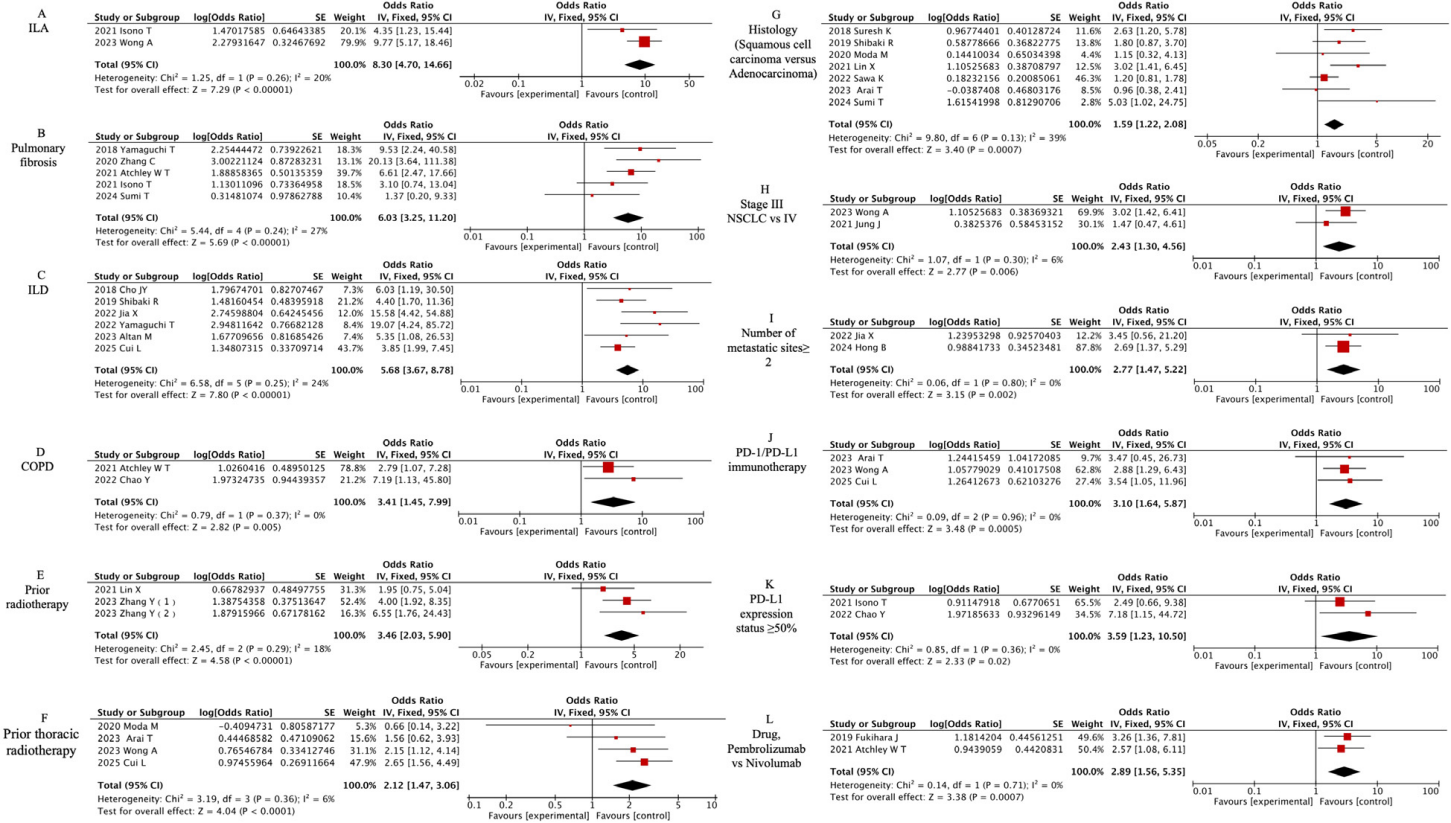
Forest plot of meta-analysis on significant clinically associated risk factors for CIP in ICI-treated lung cancer patients. **(A)** ILA, **(B)** Pulmonary fibrosis, **(C)** ILD, **(D)** COPD, **(E)** Prior radiotherapy, **(F)** Prior thoracic radiotherapy, **(G)** Histology (Squamous cell carcinoma versus Adenocarcinoma), **(H)** Stage III NSCLC vs IV, **(I)** Number of metastatic sites≥ 2, **(J)** PD-1/PD-L1 immunotherapy, **(K)** PD-L1 expression status ≥50%, **(L)** Drug, Pembrolizumab vs Nivolumab.

The meta-analysis identified 12 clinically significant predictors from 19 evaluated factors, as detailed in **Figure 4**. Preexisting pulmonary pathologies demonstrated the strongest associations: interstitial lung abnormalities (ILA) [OR=8.30, 95% CI (4.70-14.66), P<0.00001], pulmonary fibrosis (PF) [OR=6.03, 95% CI (3.25-11.2), P<0.00001], interstitial lung disease (ILD) [OR=6.03, 95% CI (3.25-11.2), P<0.00001], and chronic obstructive pulmonary disease (COPD) [OR=6.03, 95% CI (3.25-11.2), P<0.00001]. Radiation history significantly amplified CIP risk, with prior thoracic radiotherapy [OR=2.12, 95% CI (1.47-3.06), P<0.00001] and general radiotherapy history [OR=3.46, 95% CI (2.03-5.9), P<0.00001]. Tumor characteristics revealed elevated susceptibility in squamous cell carcinoma versus adenocarcinoma [OR=1.59, 95% CI (1.22-2.08), P=0.0007], stage III versus IV NSCLC [OR=2.43, 95% CI (1.30-4.56), P=0.006], and metastatic sites ≥2 [OR=2.77, 95% CI (1.47-5.22), P=0.002]. Immunotherapy comparisons showed PD-1 immunotherapy (vs PD-L1) increased risk (OR=3.10, 95% CI 1.64-5.87, P=0.0005). Pembrolizumab demonstrated higher CIP likelihood than nivolumab [OR=2.89, 95% CI (1.56-5.35), P=0.0007]. Tumors expressing PD-L1 ≥50% exhibited 3.59-fold increased risk [OR=3.59, 95% CI (1.23-10.50), P=0.02].

#### 3.3.3 Laboratory characteristics

**Figure 5:**
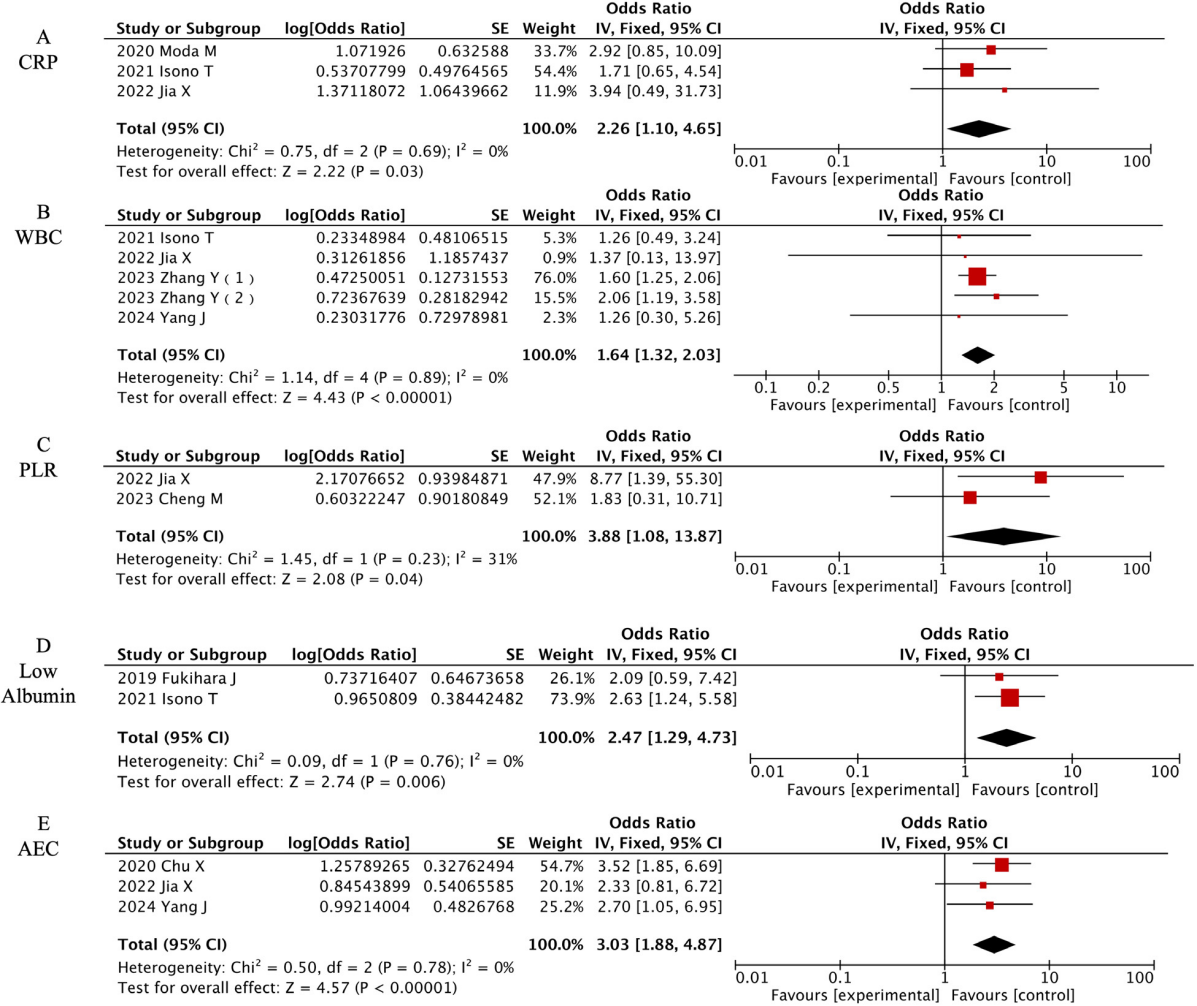
Forest plot of meta-analysis on significant laboratory-associated risk factors for CIP in ICI-treated lung cancer patients. **(A)** CRP, **(B)** WBC, **(C)** PLR, **(D)** Low Albumin, **(E)** AEC.

Five laboratory parameters demonstrated significant predictive value among eight laboratory factors, as detailed in **Figure 5**. [OR=2.26, 95% CI (1.1-4.65), P=0.03] for elevated C-reactive protein (CRP) and [OR=3.88, 95% CI (1.08-13.87), P=0.04] for elevated White Blood Cells (WBC) indicated that both inflammatory markers are risk factors for CIP. [OR=3.88, 95% CI (1.08-13.87), P=0.04] showed that a high platelet-to-lymphocyte ratio (PLR) is a risk factor for CIP, [OR=2.47, 95% CI (1.29-4.73), P=0.006] indicated that low albumin is a risk factor for CIP, and [OR=3.03, 95% CI (1.88-4.87), P<0.00001] showed that absolute eosinophil count (AEC) is a risk factor for CIP.

### 3.4 Heterogeneity analysis

The results indicated heterogeneity for the following factors: Combined treatment (combined IO/IO monotherapy) (P=0.08, I2 = 67%), ALC (P=0.002, I2 = 79%), lung diseases (P=0.01, I2 = 84%), emphysema (P=0.006, I2 = 81%), extrathoracic metastasis (P=0.03, I2 = 78%), pulmonary metastasis (P=0.003, I2 = 89%), tumor invasion in the central airway (P=0.002, I2 = 89%), and Eastern Cooperative Oncology Group performance status (ECOG PS) (≥2 versus <2) (P=0.0008, I2 = 76%). Therefore, a REM was used for these factors. For the remaining factors without heterogeneity, a FEM was applied.

Due to the limited number of included studies, further subgroup analyses based on region and sample size were not conducted for combined treatment, ALC, lung diseases, emphysema, extrathoracic metastasis, pulmonary metastasis, and tumor invasion in the central airway. Instead, REM was still used for analysis. For ECOG PS (≥2 versus <2), after excluding each study individually, the heterogeneity persisted, and the risk factor results did not change, so REM was still used for analysis.

### 3.5 Sensitivity analysis and publication bias

In addition to sensitivity analysis to identify sources of heterogeneity, this study also compared the results of FEM and REM to analyze eight factors potentially associated with CIP and estimate their combined OR values. The results in **Appendix 2** show a high degree of consistency between FEM and REM, with no reversal of results, indicating low sensitivity and further confirming the reliability of the study findings. In this study, funnel plots were created for factors with two or more studies to better assess potential publication bias. All funnel plots are provided in **Appendix 3**, showing no significant signs of publication bias.

## 4 Discussion

ICIs have revolutionized the management of advanced lung cancer by substantially improving survival outcomes, however, CIP has become a clinically significant adverse event requiring heightened vigilance. While prior investigations have attempted to delineate CIP risk factors, their findings have been somewhat contradictory. This study presents a systematic meta-analysis that integrates data across demographic characteristics, clinical features, and laboratory parameters, providing new insights into risk stratification and early intervention for lung cancer patients undergoing immunotherapy.

Our study identified demographic factors, including age, sex, and smoking history, as significant risk factors for CIP. These differences may relate to variations in immune system responsiveness and the disruption of immune balance across different populations. In elderly patients, aging is linked to thymic involution, decreased ZAP-70 phosphorylation, and diminished regulatory T cell (Treg) function, which may impair the clearance of autoreactive T cells, leading to an increased number of these cells and thereby elevating the risk of immune system attacks on the body’s tissues (45). Furthermore, sex differences play a crucial role. Research indicates that male patients exhibit higher baseline levels of inflammation, and elevated inflammatory cytokines may exacerbate immune dysregulation through inflammatory pathways, thereby increasing the risk of CIP following ICI treatment (46). Smoking, a major risk factor for lung cancer, induces chronic inflammation in the airways and alters the balance of immune cell subsets. This results in specific epigenetic modifications and dysregulated circulating T cell subsets, which can further promote the development of CIP (47). Therefore, special attention should be given to smokers during ICI therapy, with smoking cessation interventions implemented to reduce the occurrence of immune-related adverse events.

In terms of clinical characteristics, comorbidities are considered significant risk factors for the development of CIP. This study identified ILA, ILD, PF, and COPD as major risk factors for the development of CIP. These underlying pulmonary conditions are characterized by structural and functional damage, progressive pulmonary inflammation, and disruption of the immune microenvironment, as evidenced primarily by an increase in CD4^+^ T cells (33, 48). The activation of the immune system may result in attacks on already-damaged lung tissue, triggering the onset of CIP. However, while emphysema may also increase the risk of CIP, this study did not demonstrate a direct association, possibly due to the limited number of related studies. Although preexisting pulmonary comorbidities are not absolute contraindications for the use of ICIs, this finding emphasizes the clinical necessity of baseline pulmonary evaluation using pulmonary function tests (PFTs) and HRCT before starting ICI therapy, along with continuous pulmonary monitoring throughout treatment. A significant decline in parameters such as predicted FEV1% should strongly suggest the potential development of CIP. Multiple clinical guidelines, including those from the National Comprehensive Cancer Network, now recommend baseline PFTs before initiating ICIs and consider them an essential part of the diagnostic process for CIP (49). Additionally, prior treatments, such as a history of radiotherapy or thoracic radiation, are considered risk factors for CIP. Radiotherapy, by exposing tumor antigens, promotes the recognition of tumor-associated T cells and the elimination of cancer cells; however, this process may also lead to excessive immune activation, thereby inducing CIP (50, 51).

Tumor features, including PD-L1 expression, histological type, and stage, also influence the risk of CIP. Specifically, PD-L1 overexpression, histological type, and cancer staging can facilitate CIP through various immune mechanisms. This study demonstrated that high PD-L1 expression is a risk factor for the development of CIP, consistent with the findings of Chao Y et al (33). The interaction between PD-1 and its ligands PD-L1/PD-L2 serves as a negative regulator of T-cell function, maintaining the equilibrium between T-cell activation, immune tolerance, and immunologically mediated tissue damage. PD-L1 exhibits broad cellular expression patterns, including tumor cells, immune cells, and normal tissues (epithelial/endothelial cells). In physiological conditions, PD-1/PD-L1 signaling preserves immune homeostasis by suppressing T-cell activity and mitigating excessive inflammatory responses. Conversely, tumor cells exploit this pathway to evade immune surveillance. While ICIs enhance antitumor immunity, they simultaneously disrupt peripheral tolerance mechanisms, leading to collateral pulmonary damage and subsequent CIP. Higher PD-L1 expression levels correlate with increased risk of such “off-target” effects and CIP development (52). Literature reports a median CIP onset time of 3.9 months (53). Given the enhanced immune activation in PD-L1-high patients, this temporal window may shift earlier. Therefore, for PD-L1-high populations, we recommend (1): Baseline pulmonary assessment (HRCT and PFTs) before ICI initiation (2); Close monitoring of respiratory symptoms (cough/dyspnea) and radiographic features (ground-glass opacities/consolidations on chest CT) during treatment; (3) Immediate suspension of immunotherapy and prompt initiation of corticosteroid therapy upon CIP suspicion to prevent irreversible lung injury.

Squamous cell carcinoma has also been identified as a risk factor for CIP, potentially attributable to its characteristically higher PD-L1 expression compared to adenocarcinoma. Mechanistically, PD-L1 regulation involves multiple signaling molecules, including STAT family, MAPKs, IRF-1, and PIK3. The expression of STAT3 in lung squamous cell carcinoma tissue is higher than that in lung adenocarcinoma tissue (54), which may drive PD-L1 upregulation through STAT3-mediated binding to Jab1 or direct promoter activation (55), thereby enhancing pulmonary immunogenicity and predisposing to CIP pathogenesis. Studies indicate that obstructive pneumonia (POP), caused by airway stenosis, is more prone to chronic inflammation and infection, thereby increasing the risk of CIP (29). Research by Yu W et al. showed that squamous cell carcinoma accounted for 85.25% of POP cases (56). Notably, smoking-induced tumors are predominantly composed of squamous cell carcinoma. Squamous cell carcinoma originates from squamous metaplasia of the bronchial epithelium and often presents as central lung cancer, leading to airway narrowing and subsequent inflammation. Moreover, squamous cell carcinoma typically exhibits elevated PD-L1 expression, further contributing to immune system overactivation and increasing the risk of CIP.

Our study found that patients with stage III NSCLC had a significantly higher risk of CIP than those with stage IV, a conclusion consistent with the findings of Alexander Wong et al (40). Stage III NSCLC, which remains localized without distant metastasis, is associated with a higher intrathoracic tumor burden than stage IV, which has metastasized to distant organs. Previous studies suggest that patients with stage III NSCLC, characterized by a high local tumor burden, tend to experience immune cell accumulation in the lungs, leading to local immune activation and triggering CIP (38). Although our study did not confirm extrathoracic metastasis as a direct risk factor for CIP, it is plausible that the high tumor burden in stage III NSCLC predisposes patients to immune-related lung damage. Additionally, patients with stage III NSCLC are more likely to receive radiotherapy as part of combination therapy, which could further contribute to the development of CIP. However, the short survival time of patients with stage IV NSCLC limits the evaluation of long-term immune therapy effects, and further prospective studies are needed to better understand the mechanisms of CIP in both stages. Furthermore, the presence of multifocal metastases significantly increases the risk of CIP, consistent with the findings of Baohui Hong et al (35). This phenomenon is likely linked to elevated IL-6 levels at metastatic sites, as IL-6 drives both tumor metastasis and immune cell activation, establishing a positive feedback loop that culminates in cytokine storm-induced lung injury and CIP (57, 58).

The choice of ICI therapy plays a critical role in the development of CIP. Notably, there are differences in CIP incidence between anti-PD-1 and anti-PD-L1 treatments. Our study found that anti-PD-1 therapy is more likely to induce CIP compared to anti-PD-L1 therapy, which is consistent with the findings of Yin J et al (59). The variation in CIP incidence and severity may be attributed to differences in the PD-1 ligands, such as PD-L1 and PD-L2. PD-1 inhibitors block both PD-L1 and PD-L2, thereby enhancing anti-tumor immunity. However, the blockade of PD-1 disrupts the balance of PD-L2 binding to RGMb, promoting the clonal expansion of T cells in the lungs and impairing immune tolerance, thus contributing to the development of CIP (59). In contrast, PD-L1 inhibitors do not affect PD-L2. Several studies have demonstrated that patients treated with PD-1 inhibitors have a higher incidence of pneumonia compared to those treated with PD-L1 inhibitors, which supports our findings (60, 61). Although both therapies target PD-1, this study suggests that pembrolizumab may carry a higher risk of CIP compared to nivolumab, likely due to its greater ability to induce PD-1 internalization (62). However, the exact mechanisms underlying these differences remain to be fully elucidated.

Meanwhile, although heterogeneity was addressed through random-effects models, persistent heterogeneity in ECOG PS (I²=76%) remained evident. This likely reflects patient diversity inherent to real-world clinical settings. The observed variability may originate not merely from methodological variations (e.g., inter-rater variability), but more fundamentally from biological and clinical heterogeneity across study populations. For instance, the enrolled patients may have spanned different therapeutic phases from palliative care to curative-intent antitumor therapy, or included populations with significant disparities in primary tumor types, comorbidity burden, and prior treatment histories. Notably, as a dynamic clinical parameter, the association strength between ECOG PS and immune checkpoint inhibitor-related pneumonitis may exhibit nonlinear relationships contingent upon clinical thresholds of baseline functional status. Future investigations should standardize the stratification cutoffs for ECOG scoring and report longitudinal trajectories of functional status evolution to more precisely quantify the effect size of this risk factor.

In our study, five laboratory indicators were identified as risk factors for CIP. Among these, PLR was found to be a significant risk factor. PLR, which reflects systemic inflammation within the tumor microenvironment, indicates platelet hyperactivation and lymphocyte exhaustion. Platelets facilitate tumor cell invasion and metastasis by inducing pro-inflammatory cytokines, which indirectly promote immune cell recruitment to both primary and metastatic tumors. This process contributes to immune system overactivation, potentially leading to CIP (63, 64). Additionally, our research identified elevated AEC as a risk factor for CIP. Previous studies have suggested that T-cell activation and immune dysregulation are potential mechanisms underlying the pathogenesis of CIP (5). Study demonstrates that ICIs stimulate IL-5 secretion by CD4^+^ T cells, thereby enhancing eosinophil production in bone marrow and elevating peripheral blood eosinophil counts (65). These eosinophils can be recruited to tumor sites through chemotaxis mediated by type 2 cytokines (IL-5, IL-4, IL-10, and IL-13) produced by tumor cells. Mechanistically, eosinophils exert dual antitumor effects: direct cytotoxic activity via degranulation, and recruitment/activation of CD8^+^ T cells to amplify tumoricidal responses (66). Consequently, increased AEC in peripheral blood may partially reflect ICI therapeutic efficacy. However, when ICI-accumulated eosinophils infiltrate normal lung tissue, they establish a proinflammatory feedback loop through secretion of cytokines (e.g., IL-5, GM-CSF), subsequently recruiting additional immune cells (neutrophils, Th2 cells) that collectively mediate CIP pathogenesis. Critical clinical evidence suggests that an increase in eosinophils to ≥3.0% before two courses of treatment may be a practical biomarker for irAE occurrence (67). Although the determination of its threshold value is still debatable, this also suggests that high AEC can be used as a biomarker for predicting CIP occurrence in the clinical use of ICIs, and careful evaluation of the eosinophil proportion can be used for early prediction and management of CIP.

Moreover, low albumin, high CRP, and elevated WBC count are also associated with an increased risk of CIP. Although the exact mechanisms remain unclear, albumin plays a crucial role in regulating inflammation, maintaining vascular integrity, and stabilizing oncotic pressure. Hypoalbuminemia reduces osmotic pressure, leading to pulmonary edema, impaired lung microcirculation, and the release of pro-inflammatory cytokines, all of which exacerbate oxidative stress and tissue damage, thereby contributing to CIP (68). Elevated CRP and WBC reflect excessive systemic inflammatory activation, with neutrophil-derived inflammatory mediators further recruiting immune cells, establishing a positive feedback loop, and exacerbating lung injury (69). Zhang Y et al. found that the NLR, a marker of inflammatory balance, is a risk factor for CIP, though this was not confirmed in our study (19). Future prospective research is needed to clarify the role of NLR and other inflammatory markers in CIP pathogenesis.

Although laboratory parameters such as PLR, AEC, low albumin, high CRP, and WBC count show potential for predicting CIP, their prognostic or predictive utility is limited by variable results across studies. Some research has indicated inconsistent correlations between these inflammatory markers and immune response dynamics (70), suggesting that composite predictive models integrating multiple laboratory indices may offer more reliable risk stratification for CIP than relying on single biomarkers (71). Currently, no CIP-specific serological biomarkers are available in clinical practice (19). Although existing models based on these laboratory parameters show limited capacity to identify patients at significant risk for CIP, they remain valuable for early screening and ongoing monitoring. Given this, we recommend routine measurement of these parameters, particularly in high-risk populations such as patients with pre-existing pulmonary conditions or elevated PD-L1 expression. Regular monitoring of PLR, AEC, albumin, CRP, and WBC count should be integrated into clinical practice, enabling early detection of pulmonary inflammation or immune-related lung injury and facilitating timely intervention to prevent irreversible damage. Incorporating these laboratory indices into routine clinical monitoring will improve the ability to predict, detect, and manage CIP, ultimately enhancing patient outcomes and minimizing the risks associated with immune checkpoint inhibitor therapy.

An additional important consideration in this study is the variability in diagnostic criteria for CIP across the included literature. Several studies relied on radiological features from HRCT, such as ground-glass opacities with organizing pneumonia patterns, while others required histopathological confirmation. Relying solely on HRCT imaging may lead to the inclusion of asymptomatic or subclinical cases, potentially increasing the risk of false-positive diagnoses. Conversely, the requirement for pathological verification might introduce selection bias, favoring more severe cases. This variability in diagnostic approaches could have influenced the pooled analytical outcomes and represents a potential source of bias in the present investigation.

Although this study systematically assessed the core risk factors for CIP, the limited number of included studies prevented further subgroup analyses. However, it is important to emphasize that CIP risk exhibits racial disparities, with East Asian populations showing higher susceptibility compared to other ethnic groups. This disparity may be associated with genetic variations such as HLA-B35 and DRB111 (72). Additionally, even within the same comorbidities, variations in disease activity levels can occur, and differences in treatment regimens (e.g., combination chemotherapy versus monotherapy with ICIs) may influence CIP risk through modulation of the immune microenvironment. These factors—ethnicity, comorbidities, and treatment accessibility—can all potentially confound our findings, underscoring the need for caution when extrapolating the results to broader clinical settings.

In conclusion, this study identifies multiple risk factors for CIP. This comprehensive analysis contributes to the understanding of CIP risk, providing a foundation for early diagnosis and management. The innovation of this study lies in its meta-analysis of risk factors based on multivariate data, addressing limitations such as small sample sizes and insufficient laboratory parameters in previous analyses. However, this study also has several limitations (1): We fully acknowledge the inherent limitations associated with the retrospective study design, particularly the susceptibility to various biases. While we employed the NOS to rigorously select and include studies with a score of ≥7 to minimize bias, the retrospective nature of the study inherently presents challenges. In particular, controlling for potential confounders remains a complex issue, and the possibility of selection bias and information bias cannot be eliminated. Therefore, future prospective studies are essential to further substantiate and validate our findings (2); The second limitation of this study is the overrepresentation of the Asian population in the cohort, which accounts for 73% of the participants. This bias may affect the generalizability of the results, particularly about non-Asian populations. While the Asian cohort is highly representative in terms of the incidence of immune checkpoint inhibitor-related pneumonitis, future studies should place greater emphasis on racial diversity by including patients from different racial backgrounds to further validate the clinical manifestations and incidence of immune checkpoint inhibitor-related pneumonitis in other populations; (3) To improve generalizability, we excluded studies with a total sample size of <50. However, we acknowledge that this may limit our ability to detect rare risk factors that could be identified in smaller studies; (4) Despite this study systematically evaluating core risk factors for CIP, the limited number of included studies precluded further subgroup analyses; (5) There are differences in the diagnostic criteria for CIP included in this study, and most of them only explore the overall risk factors for CIP occurrence, without further exploring the specific risk factors for different levels of CIP occurrence; (6) Most of the included studies focus on PD-1/PD-L1 inhibitor-induced pneumonia, with limited data on other ICIs, such as CTLA-4 inhibitors.

## 5 Conclusion

This meta-analysis delineates 20 significant risk predictors for CIP in lung cancer patients undergoing ICI therapy. Demographic determinants include advanced age, male sex, and smoking status. Clinical predictors encompass preexisting ILA, pulmonary fibrosis, ILD, COPD, prior radiotherapy (particularly thoracic), squamous cell carcinoma histology (vs. adenocarcinoma), early-stage NSCLC (Stage III versus IV), multifocal metastases (≥2 sites), PD-1 immunotherapy (vs. PD-L1 agents), elevated PD-L1 expression (≥50%), and pembrolizumab administration (versus nivolumab). Laboratory biomarkers demonstrating predictive utility comprise AEC, CRP, PLR, WBC, and low albumin. For ICI-treated lung cancer patients with identified risk factors, implementation of risk-stratified surveillance protocols incorporating advanced imaging modalities and optimized therapeutic monitoring represents a critical strategy to reduce CIP incidence, prevent severe pulmonary complications, and improve clinical outcomes. Comprehensive management approaches should systematically combine pretreatment comorbidity assessment with dynamic biomarker evaluation to facilitate appropriate corticosteroid intervention when clinically warranted. Future investigations must prioritize large-scale prospective multicenter studies to validate and elucidate risk stratification models for CIP development in ICI-treated lung cancer populations, with particular emphasis on ethnic variability, therapeutic sequencing, and biomarker integration.

## Data Availability

The original contributions presented in the study are included in the article/**Supplementary Material**. Further inquiries can be directed to the corresponding author.

## References

[1] Bray F Laversanne M Sung H Ferlay J Siegel RL Soerjomataram I . Global cancer statistics 2022: GLOBOCAN estimates of incidence and mortality worldwide for 36 cancers in 185 countries. CA Cancer J Clin. (2024) 74:229–63. doi: 10.3322/caac.21834 38572751

[2] Hirsch FR Scagliotti GV Mulshine JL Kwon R Curran WJ Wu Y-L . Lung cancer: current therapies and new targeted treatments. Lancet. (2017) 389:299–311. doi: 10.1016/S0140-6736(16)30958-8 27574741

[3] Ribas A Wolchok JD . Cancer immunotherapy using checkpoint blockade. Science. (2018) 359:1350–5. doi: 10.1126/science.aar4060 PMC7391259 29567705

[4] Topalian SL Forde PM Emens LA Yarchoan M Smith KN Pardoll DM . Neoadjuvant immune checkpoint blockade: A window of opportunity to advance cancer immunotherapy. Cancer Cell. (2023) 41:1551–66. doi: 10.1016/j.ccell.2023.07.011 PMC10548441 37595586

[5] Ghanbar MI Suresh K . Pulmonary toxicity of immune checkpoint immunotherapy. J Clin Invest. (2024) 134:e170503. doi: 10.1172/JCI170503 38226621 PMC10786690

[6] Zhai X Zhang J Tian Y Li J Jing W Guo H . The mechanism and risk factors for immune checkpoint inhibitor pneumonitis in non-small cell lung cancer patients. Cancer Biol Med. (2020) 17:599–611. doi: 10.20892/j.issn.2095-3941.2020.0102 32944393 PMC7476083

[7] Wang Y Zhang T Huang Y Li W Zhao J Yang Y . Real-world safety and efficacy of consolidation durvalumab after chemoradiation therapy for stage III non-small cell lung cancer: A systematic review and meta-analysis. Int J Radiat Oncol Biol Phys. (2022) 112:1154–64. doi: 10.1016/j.ijrobp.2021.12.150 34963558

[8] Puzanov I Diab A Abdallah K Bingham CO Brogdon C Dadu R . Managing toxicities associated with immune checkpoint inhibitors: consensus recommendations from the Society for Immunotherapy of Cancer (SITC) Toxicity Management Working Group. J Immunother Cancer. (2017) 5:95. doi: 10.1186/s40425-017-0300-z 29162153 PMC5697162

[9] Wang DY Salem J-E Cohen JV Chandra S Menzer C Ye F . Fatal toxic effects associated with immune checkpoint inhibitors: A systematic review and meta-analysis. JAMA Oncol. (2018) 4:1721–8. doi: 10.1001/jamaoncol.2018.3923 PMC6440712 30242316

[10] Cadranel J Canellas A Matton L Darrason M Parrot A Naccache J-M . Pulmonary complications of immune checkpoint inhibitors in patients with nonsmall cell lung cancer. Eur Respir Rev. (2019) 28:190058. doi: 10.1183/16000617.0058-2019 31597674 PMC9488121

[11] Lin M-X Zang D Liu C-G Han X Chen J . Immune checkpoint inhibitor-related pneumonitis: research advances in prediction and management. Front Immunol. (2024) 15:1266850. doi: 10.3389/fimmu.2024.1266850 38426102 PMC10902117

[12] Nishino M Giobbie-Hurder A Hatabu H Ramaiya NH Hodi FS . Incidence of programmed cell death 1 inhibitor-related pneumonitis in patients with advanced cancer: A systematic review and meta-analysis. JAMA Oncol. (2016) 2:1607–16. doi: 10.1001/jamaoncol.2016.2453 27540850

[13] Naidoo J Wang X Woo KM Iyriboz T Halpenny D Cunningham J . Pneumonitis in patients treated with anti-programmed death-1/programmed death ligand 1 therapy. J Clin Oncol. (2017) 35:709–17. doi: 10.1200/JCO.2016.68.2005 PMC5559901 27646942

[14] Cui P Liu Z Wang G Ma J Qian Y Zhang F . Risk factors for pneumonitis in patients treated with anti-programmed death-1 therapy: A case-control study. Cancer Med. (2018) 7:4115–20. doi: 10.1002/cam4.1579 PMC6089164 29797416

[15] Teijeira L Martínez M Moreno A de Elejoste I Ibáñez-Beroiz B Arrazubi V . Baseline circulating blood cell counts and ratios and changes therein for predicting immune-related adverse events during immune checkpoint inhibitor therapy: A multicenter, prospective, observational, pan-cancer cohort study with a gender perspective. Cancers (Basel). (2023) 16:151. doi: 10.3390/cancers16010151 38201577 PMC10778233

[16] Page MJ Moher D Bossuyt PM Boutron I Hoffmann TC Mulrow CD . PRISMA 2020 explanation and elaboration: updated guidance and exemplars for reporting systematic reviews. BMJ. (2021) 372:n160. doi: 10.1136/bmj.n160 33781993 PMC8005925

[17] Suresh K Voong KR Shankar B Forde PM Ettinger DS Marrone KA . Pneumonitis in non-small cell lung cancer patients receiving immune checkpoint immunotherapy: incidence and risk factors. J Thorac Oncol. (2018) 13:1930–9. doi: 10.1016/j.jtho.2018.08.2035 30267842

[18] Cheng M Lin R Bai N Zhang Y Wang H Guo M . Deep learning for predicting the risk of immune checkpoint inhibitor-related pneumonitis in lung cancer. Clin Radiol. (2023) 78:e377–85. doi: 10.1016/j.crad.2022.12.013 36914457

[19] Zhang Y Zhang L Cao S Wang Y Ling X Zhou Y . A nomogram model for predicting the risk of checkpoint inhibitor-related pneumonitis for patients with advanced non-small-cell lung cancer. Cancer Med. (2023) 12:15998–6010. doi: 10.1002/cam4.6244 PMC10469710 37409360

[20] Jia X Chu X Jiang L Li Y Zhang Y Mao Z . Predicting checkpoint inhibitors pneumonitis in non-small cell lung cancer using a dynamic online hypertension nomogram. Lung Cancer. (2022) 170:74–84. doi: 10.1016/j.lungcan.2022.06.001 35717705

[21] Yamaguchi T Shimizu J Oya Y Watanabe N Hasegawa T Horio Y . Risk factors for pneumonitis in patients with non-small cell lung cancer treated with immune checkpoint inhibitors plus chemotherapy: A retrospective analysis. Thorac Cancer. (2022) 13:724–31. doi: 10.1111/1759-7714.14308 PMC8888158 35044093

[22] Wang H Zhou F Zhao C Cheng L Zhou C Qiao M . Interleukin-10 is a promising marker for immune-related adverse events in patients with non-small cell lung cancer receiving immunotherapy. Front Immunol. (2022) 13:840313. doi: 10.3389/fimmu.2022.840313 35222434 PMC8863608

[23] Isono T Kagiyama N Takano K Hosoda C Nishida T Kawate E . Outcome and risk factor of immune-related adverse events and pneumonitis in patients with advanced or postoperative recurrent non-small cell lung cancer treated with immune checkpoint inhibitors. Thorac Cancer. (2021) 12:153–64. doi: 10.1111/1759-7714.13736 PMC7812074 33201587

[24] Chu X Zhao J Zhou J Zhou F Jiang T Jiang S . Association of baseline peripheral-blood eosinophil count with immune checkpoint inhibitor-related pneumonitis and clinical outcomes in patients with non-small cell lung cancer receiving immune checkpoint inhibitors. Lung Cancer. (2020) 150:76–82. doi: 10.1016/j.lungcan.2020.08.015 33080551

[25] Moda M Saito H Kato T Usui R Kondo T Nakahara Y . Tumor invasion in the central airway is a risk factor for early-onset checkpoint inhibitor pneumonitis in patients with non-small cell lung cancer. Thorac Cancer. (2020) 11:3576–84. doi: 10.1111/1759-7714.13703 PMC7705619 33078531

[26] Fukihara J Sakamoto K Koyama J Ito T Iwano S Morise M . Prognostic impact and risk factors of immune-related pneumonitis in patients with non-small-cell lung cancer who received programmed death 1 inhibitors. Clin Lung Cancer. (2019) 20:442–450.e4. doi: 10.1016/j.cllc.2019.07.006 31446020

[27] Zhang C Gao F Jin S Gao W Chen S Guo R . Checkpoint inhibitor pneumonitis in Chinese lung cancer patients: clinical characteristics and risk factors. Ann Palliat Med. (2020) 9:3957–65. doi: 10.21037/apm-20-1823 33302658

[28] Lin X Deng H Yang Y Wu J Qiu G Li S . Peripheral blood biomarkers for early diagnosis, severity, and prognosis of checkpoint inhibitor-related pneumonitis in patients with lung cancer. Front Oncol. (2021) 11:698832. doi: 10.3389/fonc.2021.698832 34327140 PMC8313853

[29] Atchley WT Alvarez C Saxena-Beem S Schwartz TA Ishizawar RC Patel KP . Immune checkpoint inhibitor-related pneumonitis in lung cancer: real-world incidence, risk factors, and management practices across six health care centers in north carolina. Chest. (2021) 160:731–42. doi: 10.1016/j.chest.2021.02.032 PMC8411447 33621599

[30] Altan M Soto F Zhong LL Akhmedzhanov FO Wilson NR Zarifa A . Incidence and risk factors for pneumonitis associated with checkpoint inhibitors in advanced non-small cell lung cancer: A single center experience. Oncologist. (2023) 28:e1065–74. doi: 10.1093/oncolo/oyad118 PMC10628566 37156009

[31] Yang J Lyu M Feng X Liu F Zeng R Sun X . The predict factors and clinical prognosis value of immune-related pneumonia of receiving PD-1 inhibitor in advanced non-small cell lung cancer: A retrospective study. Int Immunopharmacol. (2024) 142:113140. doi: 10.1016/j.intimp.2024.113140 39312858

[32] Sumi T Sekikawa M Koshino Y Nagayama D Nagahisa Y Matsuura K . Risk factors for severe immune-related pneumonitis after nivolumab plus ipilimumab therapy for non-small cell lung cancer. Thorac Cancer. (2024) 15:1572–81. doi: 10.1111/1759-7714.15385 PMC11246787 38828610

[33] Chao Y Zhou J Hsu S Ding N Li J Zhang Y . Risk factors for immune checkpoint inhibitor-related pneumonitis in non-small cell lung cancer. Transl Lung Cancer Res. (2022) 11:295–306. doi: 10.21037/tlcr-22-72 35280322 PMC8902097

[34] Cui L Cheng K Cui M Li X . Characteristics and risk factors of immune checkpoint inhibitor-related pneumonitis in non-small cell lung cancer: A retrospective study. Oncology. (2025), 1–18. doi: 10.1159/000543556 39929164

[35] Hong B Chen R Zheng C Liu M Yang J . Development and validation of a nomogram for predicting immune-related pneumonitis after sintilimab treatment. Cancer Med. (2024) 13:e6708. doi: 10.1002/cam4.6708 38214102 PMC10905226

[36] Li X Yang F Liu B Ye L Du J Fan X . Clinical manifestation, risk factors, and immune checkpoint inhibitor rechallenge of checkpoint inhibitor-associated pneumonitis in patients with lung cancer. J Immunother. (2024) 47:220–6. doi: 10.1097/CJI.0000000000000515 38618919

[37] Shibaki R Murakami S Matsumoto Y Yoshida T Goto Y Kanda S . Association of immune-related pneumonitis with the presence of preexisting interstitial lung disease in patients with non-small lung cancer receiving anti-programmed cell death 1 antibody. Cancer Immunol Immunother. (2020) 69:15–22. doi: 10.1007/s00262-019-02431-8 31745589 PMC11027879

[38] Cho JY Kim J Lee JS Kim YJ Kim SH Lee YJ . Characteristics, incidence, and risk factors of immune checkpoint inhibitor-related pneumonitis in patients with non-small cell lung cancer. Lung Cancer. (2018) 125:150–6. doi: 10.1016/j.lungcan.2018.09.015 30429014

[39] Yamaguchi T Shimizu J Hasegawa T Horio Y Inaba Y Yatabe Y . Pre-existing pulmonary fibrosis is a risk factor for anti-PD-1-related pneumonitis in patients with non-small cell lung cancer: A retrospective analysis. Lung Cancer. (2018) 125:212–7. doi: 10.1016/j.lungcan.2018.10.001 30429022

[40] Wong A Riley M Zhao S Zimmer J Viveiros M Wang JG . Association between pretreatment chest imaging and immune checkpoint inhibitor pneumonitis among patients with lung cancer. J Natl Compr Canc Netw. (2023) 21:1164–1171.e5. doi: 10.6004/jnccn.2023.7059 37935100

[41] Sawa K Sato I Takeuchi M Kawakami K . Risk of pneumonitis in non-small cell lung cancer patients with preexisting interstitial lung diseases treated with immune checkpoint inhibitors: a nationwide retrospective cohort study. Cancer Immunol Immunother. (2023) 72:591–8. doi: 10.1007/s00262-022-03281-7 PMC10991202 35994088

[42] Jung J Kim HY Kim D-G Park SY Ko AR Han J-Y . Sequential treatment with an immune checkpoint inhibitor followed by a small-molecule targeted agent increases drug-induced pneumonitis. Cancer Res Treat. (2021) 53:77–86. doi: 10.4143/crt.2020.543 32777877 PMC7812016

[43] Osaki M Arai T Sumikawa H Takimoto T Takeuchi N Tamiya A . Immune checkpoint inhibitor-related pneumonitis in lung cancer patients with interstitial lung disease: significance of radiological pleuroparenchymal fibroelastosis. Oncology. (2023) 101:303–12. doi: 10.1159/000529204 36689929

[44] Li Y Jiang Y Pan L Yao J Liang S Du Y . First-line chemoimmunotherapy for patients with small-cell lung cancer and interstitial lung abnormality: CIP risk and prognostic analysis. Thorac Cancer. (2024) 15:2437–48. doi: 10.1111/1759-7714.15471 PMC11609049 39435523

[45] Müller L Di Benedetto S . From aging to long COVID: exploring the convergence of immunosenescence, inflammaging, and autoimmunity. Front Immunol. (2023) 14:1298004. doi: 10.3389/fimmu.2023.1298004 37942323 PMC10628127

[46] Islam H Jackson GS Yoon JSJ Cabral-Santos C Lira FS Mui AL . Sex differences in IL-10’s anti-inflammatory function: greater STAT3 activation and stronger inhibition of TNF-α production in male blood leukocytes ex vivo. Am J Physiol Cell Physiol. (2022) 322:C1095–104. doi: 10.1152/ajpcell.00091.2022 35508192

[47] Saint-André V Charbit B Biton A Rouilly V Possémé C Bertrand A . Smoking changes adaptive immunity with persistent effects. Nature. (2024) 626:827–35. doi: 10.1038/s41586-023-06968-8 PMC10881394 38355791

[48] Lee KS Han J Wada N Hata A Lee HY Yi C . Imaging of pulmonary fibrosis: an update, from the AJR special series on imaging of fibrosis. AJR Am J Roentgenol. (2024) 222:e2329119. doi: 10.2214/AJR.23.29119 37095673

[49] Reuss JE Brigham E Psoter KJ Voong KR Shankar B Ettinger DS . Pretreatment lung function and checkpoint inhibitor pneumonitis in NSCLC. JTO Clin Res Rep. (2021) 2:100220. doi: 10.1016/j.jtocrr.2021.100220 34746881 PMC8552105

[50] Martinov T Fife BT . Fractionated radiotherapy combined with PD-1 pathway blockade promotes CD8 T cell-mediated tumor clearance for the treatment of advanced Malignancies. Ann Transl Med. (2016) 4:82. doi: 10.3978/j.issn.2305-5839.2016.01.13 27004229 PMC4779769

[51] Sharabi AB Lim M DeWeese TL Drake CG . Radiation and checkpoint blockade immunotherapy: radiosensitisation and potential mechanisms of synergy. Lancet Oncol. (2015) 16:e498–509. doi: 10.1016/S1470-2045(15)00007-8 26433823

[52] Li X Shao C Shi Y Han W . Lessons learned from the blockade of immune checkpoints in cancer immunotherapy. J Hematol Oncol. (2018) 11:31. doi: 10.1186/s13045-018-0578-4 29482595 PMC6389077

[53] Tiu BC Zubiri L Iheke J Pahalyants V Theodosakis N Ugwu-Dike P . Real-world incidence and impact of pneumonitis in patients with lung cancer treated with immune checkpoint inhibitors: a multi-institutional cohort study. J Immunother Cancer. (2022) 10:e004670. doi: 10.1136/jitc-2022-004670 35705313 PMC9204442

[54] Liu LU Xie B Zhu W He Q Zhou J Liu S . High expression of PD-L1 mainly occurs in non-small cell lung cancer patients with squamous cell carcinoma or poor differentiation. Oncol Res. (2023) 31:275–86. doi: 10.32604/or.2023.028227 PMC10229306 37305382

[55] Zhu P Jin Z Kang G Jia Y Liu D Zhang Q . Alpha5 nicotinic acetylcholine receptor mediated immune escape of lung adenocarcinoma via STAT3/Jab1-PD-L1 signalling. Cell Commun Signal. (2022) 20:121. doi: 10.1186/s12964-022-00934-z 35971127 PMC9377093

[56] Yu W Shi Y Zheng Q Chen J Zhang X Chen A . Comparison between community-acquired pneumonia and post-obstructive pneumonia associated with endobronchial tumors. BMC Pulm Med. (2024) 24:589. doi: 10.1186/s12890-024-03409-8 39609797 PMC11606229

[57] Werner-Klein M Grujovic A Irlbeck C Obradović M Hoffmann M Koerkel-Qu H . Interleukin-6 trans-signaling is a candidate mechanism to drive progression of human DCCs during clinical latency. Nat Commun. (2020) 11:4977. doi: 10.1038/s41467-020-18701-4 33020483 PMC7536220

[58] Jones SA Jenkins BJ . Recent insights into targeting the IL-6 cytokine family in inflammatory diseases and cancer. Nat Rev Immunol. (2018) 18:773–89. doi: 10.1038/s41577-018-0066-7 30254251

[59] Yin J Wu Y Yang X Gan L Xue J . Checkpoint inhibitor pneumonitis induced by anti-PD-1/PD-L1 therapy in non-small-cell lung cancer: occurrence and mechanism. Front Immunol. (2022) 13:830631. doi: 10.3389/fimmu.2022.830631 35464480 PMC9021596

[60] Khunger M Rakshit S Pasupuleti V Hernandez AV Mazzone P Stevenson J . Incidence of pneumonitis with use of programmed death 1 and programmed death-ligand 1 inhibitors in non-small cell lung cancer: A systematic review and meta-analysis of trials. Chest. (2017) 152:271–81. doi: 10.1016/j.chest.2017.04.177 28499515

[61] Pillai RN Behera M Owonikoko TK Kamphorst AO Pakkala S Belani CP . Comparison of the toxicity profile of PD-1 versus PD-L1 inhibitors in non-small cell lung cancer: A systematic analysis of the literature. Cancer. (2018) 124:271–7. doi: 10.1002/cncr.31043 PMC5761314 28960263

[62] Zhang Y Wei R Song G Yang X Zhang M Liu W . Insights into the mechanisms of serplulimab: a distinctive anti-PD-1 monoclonal antibody, in combination with a TIGIT or LAG3 inhibitor in preclinical tumor immunotherapy studies. MAbs. (2024) 16:2419838. doi: 10.1080/19420862.2024.2419838 39497266 PMC11540081

[63] Coupland LA Hindmarsh EJ Gardiner EE Parish CR . The influence of platelet membranes on tumour cell behaviour. Cancer Metastasis Rev. (2017) 36:215–24. doi: 10.1007/s10555-017-9671-3 28707200

[64] Olsson AK Cedervall J . The pro-inflammatory role of platelets in cancer. Platelets. (2018) 29:569–73. doi: 10.1080/09537104.2018.1453059 29584534

[65] Blomberg OS Spagnuolo L Garner H Voorwerk L Isaeva OI van Dyk E . IL-5-producing CD4+ T cells and eosinophils cooperate to enhance response to immune checkpoint blockade in breast cancer. Cancer Cell. (2023) 41:106–123.e10. doi: 10.1016/j.ccell.2022.11.014 36525971

[66] Sibille A Corhay J-L Louis R Ninane V Jerusalem G Duysinx B . Eosinophils and lung cancer: from bench to bedside. Int J Mol Sci. (2022) 23:5066. doi: 10.3390/ijms23095066 35563461 PMC9101877

[67] Brahmer JR Lacchetti C Schneider BJ Atkins MB Brassil KJ Caterino JM . Management of immune-related adverse events in patients treated with immune checkpoint inhibitor therapy: american society of clinical oncology clinical practice guideline. J Clin Oncol. (2018) 36:1714–68. doi: 10.1200/JCO.2017.77.6385 PMC6481621 29442540

[68] Zhou K Lu J . Progress in cytokine research for ARDS: A comprehensive review. Open Med (Wars). (2024) 19:20241076. doi: 10.1515/med-2024-1076 39479463 PMC11524396

[69] Liu C Zhou R Chen B Yan X Guo L Tang Y . Inflammatory microenvironment-responsive nanomicelles for acute lung injury therapy: ROS-scavenging and macrophage repolarization. Mater Today Bio. (2025) 31:101622. doi: 10.1016/j.mtbio.2025.101622 PMC11919404 40104650

[70] Wu M Liu J Wu S Liu J Wu H Yu J . Systemic immune activation and responses of irradiation to different metastatic sites combined with immunotherapy in advanced non-small cell lung cancer. Front Immunol. (2021) 12:803247. doi: 10.3389/fimmu.2021.803247 34970277 PMC8712862

[71] Rugambwa TK Abdihamid O Zhang X Peng Y Cai C Shen H . Neutrophil-lymphocyte ratio and platelet-lymphocyte ratio as potential predictive markers of treatment response in cancer patients treated with immune checkpoint inhibitors: a systematic review and meta-analysis. Front Oncol. (2023) 13:1181248. doi: 10.3389/fonc.2023.1181248 38023176 PMC10646751

[72] Jiang N Yu Y Zhang M Tang Y Wu D Wang S . Association between germ-line HLA and immune-related adverse events. Front Immunol. (2022) 13:952099. doi: 10.3389/fimmu.2022.952099 36177028 PMC9513190

