# Supplementary material for: Risk factors for checkpoint inhibitor pneumonitis in lung cancer patients treated with immune checkpoint inhibitors: a systematic review and meta-analysis
Source: Front Immunol. 2025 May 21;16:1607170. doi: 10.3389/fimmu.2025.1607170 (PMC12133883; doi:10.3389/fimmu.2025.1607170)
Supplement: Supplementary file 1 [file DataSheet1.docx]

**Appendix 1. Search strategy in PubMed Database.**

| Number | Search terms |
| --- | --- |
| #1 | "Lung Neoplasms "[Mesh] |
| #2 | ((((((((((((((((Neoplasms, Pulmonary[Title/Abstract]) OR (Neoplasm, Pulmonary[Title/Abstract])) OR (Pulmonary Neoplasm[Title/Abstract])) OR (Pulmonary Neoplasms[Title/Abstract])) OR (Neoplasms, Lung[Title/Abstract])) OR (Lung Neoplasm[Title/Abstract])) OR (Neoplasm, Lung[Title/Abstract])) OR (Lung Cancer[Title/Abstract])) OR (Cancer, Lung[Title/Abstract])) OR (Cancers, Lung[Title/Abstract])) OR (Lung Cancers[Title/Abstract])) OR (Cancer of Lung[Title/Abstract])) OR (Pulmonary Cancer[Title/Abstract])) OR (Cancer, Pulmonary[Title/Abstract])) OR (Cancers, Pulmonary[Title/Abstract])) OR (Pulmonary Cancers[Title/Abstract])) OR (Cancer of the Lung[Title/Abstract]) |
| #3 | #1 OR #2 |
| #4 | " Immune Checkpoint Inhibitors "[Mesh] |
| #5 | ((((((((((((((((((((((((((((((((Checkpoint Inhibitors, Immune[Title/Abstract]) OR (Immune Checkpoint Blockers[Title/Abstract])) OR (Checkpoint Blockers, Immune[Title/Abstract])) OR (Immune Checkpoint Inhibitor[Title/Abstract])) OR (Checkpoint Inhibitor, Immune[Title/Abstract])) OR (CTLA-4 Inhibitors CTLA-4[Title/Abstract])) OR (CTLA 4 Inhibitors[Title/Abstract])) OR (Cytotoxic T-Lymphocyte-Associated Protein 4 Inhibitors[Title/Abstract])) OR (Cytotoxic T Lymphocyte Associated Protein 4 Inhibitors[Title/Abstract])) OR (Cytotoxic T-Lymphocyte-Associated Protein 4 Inhibitor[Title/Abstract])) OR (Cytotoxic T Lymphocyte Associated Protein 4 Inhibitor[Title/Abstract])) OR (CTLA-4 Inhibitor[Title/Abstract])) OR (CTLA 4 Inhibitor[Title/Abstract])) OR (PD-1 Inhibitors[Title/Abstract])) OR (PD 1 Inhibitors[Title/Abstract])) OR (Programmed Cell Death Protein 1 Inhibitor[Title/Abstract])) OR (Programmed Cell Death Protein 1 Inhibitors[Title/Abstract])) OR (PD-1 Inhibitor[Title/Abstract])) OR (PD-1 Inhibitor[Title/Abstract])) OR (PD 1 Inhibitor[Title/Abstract])) OR (Immune Checkpoint Blockade[Title/Abstract])) OR (Checkpoint Blockade, Immune[Title/Abstract])) OR (Immune Checkpoint Inhibition[Title/Abstract])) OR (Checkpoint Inhibition, Immune[Title/Abstract])) OR (PD-L1 Inhibitors[Title/Abstract])) OR (PD L1 Inhibitors[Title/Abstract])) OR (Programmed Death-Ligand 1 Inhibitors[Title/Abstract])) OR (Programmed Death Ligand 1 Inhibitor[Title/Abstract])) OR (PD-L1 Inhibitor[Title/Abstract])) OR (PD L1 Inhibitor[Title/Abstract])) OR (PD-1-PD-L1 Blockade[Title/Abstract])) OR (Blockade, PD-1-PD-L1[Title/Abstract])) OR (PD 1 PD L1 Blockade[Title/Abstract]) |
| #6 | #4 OR #5 |
| #7 | "Pneumonia "[Mesh] |
| #8 | ((((((((((((((((((Factor, Risk[Title/Abstract]) ) undefined (Risk Factor[Title/Abstract])) OR (Population at Risk[Title/Abstract])) OR (Populations at Risk[Title/Abstract])) OR (Risk Scores[Title/Abstract])) OR (Risk Score[Title/Abstract])) OR (Score, Risk[Title/Abstract])) OR (Risk Factor Scores[Title/Abstract])) OR (Risk Factor Score[Title/Abstract])) OR (Score, Risk Factor[Title/Abstract])) OR (Health Correlates[Title/Abstract])) OR (Correlates, Health[Title/Abstract])) OR (Social Risk Factors[Title/Abstract])) OR (Factor, Social Risk[Title/Abstract])) OR (Factors, Social Risk[Title/Abstract])) OR (Risk Factor, Social[Title/Abstract])) OR (Risk Factors, Social[Title/Abstract])) OR (Social Risk Factor[Title/Abstract]) |
| #9  #10  #11 | #7 OR #8  "Risk Factors"[Mesh]  ((((((((((((((((((Factor, Risk[Title/Abstract]) ) undefined (Risk Factor[Title/Abstract])) OR (Population at Risk[Title/Abstract])) OR (Populations at Risk[Title/Abstract])) OR (Risk Scores[Title/Abstract])) OR (Risk Score[Title/Abstract])) OR (Score, Risk[Title/Abstract])) OR (Risk Factor Scores[Title/Abstract])) OR (Risk Factor Score[Title/Abstract])) OR (Score, Risk Factor[Title/Abstract])) OR (Health Correlates[Title/Abstract])) OR (Correlates, Health[Title/Abstract])) OR (Social Risk Factors[Title/Abstract])) OR (Factor, Social Risk[Title/Abstract])) OR (Factors, Social Risk[Title/Abstract])) OR (Risk Factor, Social[Title/Abstract])) OR (Risk Factors, Social[Title/Abstract])) OR (Social Risk Factor[Title/Abstract]) |
| #12  #13 | #10 OR #11  #3 AND #6 AND #9 AND #12 |

**Appendix 2. Comparison of calculation results s FEM and REM to 20 independent risk factors for CIP in ICI-treated lung cancer patients.**

| **Risk factors** | **OR(FEM combined)** | **OR(REM combined)** |
| --- | --- | --- |
| **Demographic features** |  |  |
| Age | 1.07 | 1.07 |
| Sex (male versus famale) | 1.41 | 1.41 |
| Smoking status | 1.92 | 1.92 |
| **Clinical features** |  |  |
| pulmonary fibrosis | 6.03 | 6.03 |
| ILD | 5.68 | 5.68 |
| COPD | 3.41 | 3.41 |
| ILA | 8.30 | 8.30 |
| Prior radiotherapy | 3.46 | 3.46 |
| prior thoracic radiotherapy | 2.12 | 2.12 |
| Histology（Squamous cell carcinoma versus Adenocarcinoma） | 1.59 | 1.59 |
| Stage III NSCLC vs IV | 2.43 | 2.43 |
| Number of metastatic sites≥ 2 | 2.77 | 2.77 |
| PD-1/PD-L1 immunotherapy | 3.10 | 3.10 |
| PD-L1 expression status ≥50% | 3.59 | 3.59 |
| Drug, pembrolizumab vs Nivolumab | 2.89 | 2.89 |
| **Laboratory findings** |  |  |
| AEC | 3.03 | 3.03 |
| CRP | 2.26 | 2.26 |
| PLR | 3.88 | 3.88 |
| WBC | 1.64 | 1.64 |
| low Albumin | 2.47 | 2.47 |

**Appendix 3. Funnel plot of of meta-analysis for CIP in ICI-treated lung cancer patients.**

(2) Sex (male versus famale)

(1) Age

(4) ECOG PS (≥2 versus <2)

(3) Smoking status

(6) Stage III NSCLC vs IV

(5) Histology (Squamous cell carcinoma versus Adenocarcinoma)

(8) Number of metastatic sites≥ 2

(7) Tumor invasion in the central airway

(10) Extrathoracic metastasis

(9) Pulmonary metastasis

(12) Emphysema

(11) ILD

(14) Lung diseases

(13) Pulmonary fibrosis

(16) ILA

(15) COPD

(17) Prior thoracic radiotherapy

(18) Prior radiotherapy

(20) ALC

(19) AEC

(22) CRP

(21) ANC

(24) PLR

(23) NLR

(26) Low Albumin

(25) WBC

(28) PD-L1 expression status ≥50%

(27) PD-1/PD-L1 immunotherapy

(30) Combined treatment (Combined IO/IO monotherapy)

(29) Drug, Pembrolizumab vs Nivolumab
